# Clinical and Basic Evaluation of the Effects of Upregulated TNFAIP3 Expression on Colorectal Cancer

**DOI:** 10.1155/2022/1263530

**Published:** 2022-08-18

**Authors:** Jinhe Li, Shuang Ren, Yuhua Zhang, Bingbing Wu, Meng He, Zhen Shan, Yang Liu, Yuejing Wang

**Affiliations:** ^1^Department of General Surgery, Qiqihar First Hospital, Qiqihar 161006, China; ^2^Department of Pathology, The Affiliated Drum Tower Hospital of Nanjing University Medical School, Nanjing 210000, China; ^3^Department of Embryology, Qiqihar Medical College, Qiqihar 161006, China

## Abstract

**Objective:**

To assess the TNFAIP3 and nuclear factor *κ*B (NF*κ*B) protein expressions in colorectal cancer (CRC) tissue and to analyze the association of these proteins with the clinical pathological characteristics of CRC.

**Methods:**

The following methods should be used in clinical trials: information collection and immunohistochemical methods. The following methods are used for cell experiment: cell transfection, CCK8 detection method, transwell experiment, and western blot experiment. Explore the TNFAIP3 expression in CRC cells, and assess the effect of upregulated TNFAIP3 expression on CRC cell proliferation, invasion, and migration. In clinical experiment, we selected the tumor tissues of 39 CRC patients as our experimental samples. We also collected corresponding patient demographics, such as sex, age, cell differentiation, tumor type, and lymph node metastasis. We also analyzed the TNFAIP3 and NF*κ*B protein expressions in 20 experimental and 20 control samples and evaluated potential correlations between these two proteins and clinical pathological characteristics of CRC. For basic experiment, we established CRC cell lines with elevated TNFAIP3 expression and then randomly divided the cells into three groups, namely, TNFAIP3, NS, and Con groups. Using the transwell and CCK8 methods, we detected the CRC migration abilities and cell proliferation, respectively. We also employed western blot analysis to assess protein expression in the three groups.

**Results:**

NF*κ*B was highly expressed, and TNFAIP3 was scarcely expressed in the experimental group versus control. The expression of both these proteins were strongly related to the degree of tumor differentiation (*P* < 0.05). The TNFAIP3 and NF*κ*B protein expressions were significantly associated with lymph node metastasis and tumor differentiation (*P* < 0.05). For basic experiment, compared to the Con and NS groups, TNFAIP3 protein expression levels, cell proliferation, invasion, and migration were significantly increased in the TNFAIP3 group (*P* < 0.05).

**Conclusion:**

TNFAIP3 overexpression strongly inhibited CRC proliferation, invasion, and migration. Enhanced NF*κ*B protein expression in CRC tissues was associated with elevated malignant degree, metastasis, and TNFAIP3 protein expression in patients who demonstrated high malignant degree and metastasis. Our evidences suggest the promising potential of utilizing TNFAIP3 and NF*κ*B as important reference indices for determining the prognostic outcome of CRC. Furthermore, we revealed that TNFAIP3 overexpression inhibited CRC cell proliferation, invasion, and migration.

## 1. Introduction

Colorectal cancer (CRC) is one of the most common clinical malignancies, and it is associated with advanced metastasis, poor survival, and markedly enhanced mortality rate. In the past two decades, the incidence rate and mortality of colorectal cancer in China have shown a sharp upward trend. Colorectal cancer has made progress in surgical treatment and chemotherapy. However, recurrence of colorectal cancer is common in most patients. Therefore, the treatment effect is still not ideal [1–3]. Tumor occurrence and development is a complex biological process, with a variety of associated genes like zinc finger protein (TNFAIP3) and nuclear factor *κ*B protein, which play an essential role in tumor occurrence and development [4, 5]. Being a member of the largest family of transcription factors in mammals, the zinc finger protein family is involved in a variety of biological functions such as gene transcriptional modulation and DNA damage repair. However, the TNFAIP3 role in CRC is rarely reported. In this paper, the immunohistochemical shortcut method was used to detect the expressions of TNFAIP3 and nuclear factor *κ*B protein (NF*κ*B) proteins and assess potential relationships between the expression of these proteins and the clinical-pathological characteristics of CRC.

In recent years, with the deepening of knowledge regarding the pathogenesis of tumor research, targeted therapy for patients with advanced CRC is gaining more and more momentum. Therefore, this study aimed to explore the TNFAIP3 expression and role in CRC tissues and cells. We specifically explored the outcome of upregulated TNFAIP3 expression on CRC cell proliferation, invasion, and migration.

## 2. Clinical Experiment: Data and Methods

### 2.1. General Information

We recruited 39 CRC patients, who received radical surgery, in our hospital between March 2015 and January 2021. All patients were diagnosed with CRC via pathological diagnosis upon surgery, and all selected patients had complete medical records, and received no chemo- or radiotherapy prior to surgery. The patient ages ranged from 31 to 85 years, with a mean age of (62.2). Among them, 21 were males, and 18 were females. We employed the American Joint Committee on Cancer (AJCC)/International Cancer Alliance (UICC) criteria to determine the TNM stage of CRC (namely, stages I, II, III, and IV). The tumor diameters were between 1 and 6 cm. The degrees of tumor tissue differentiation were classified as low differentiation, intermediate differentiation, and high differentiation. The depth of tumor invasion and lymph node metastasis were also assessed. Fresh tumor tissue, as well as adjacent noncancerous tissue (over 10 cm from the edge of the tumor lesion, and no cancerous tissue infiltration was confirmed by microscopic diagnosis), were surgically removed from CRC patients. The tissue specimens were then paraffin embedded for further analysis.

### 2.2. Equipment and Reagents

Main equipment: ordinary optical microscope (Leica, Germany); inverted microscope (Leica, Germany); and immunohistochemical detection kit and DAB developer (BOSTER, China). The rabbit antihuman TNFAIP3 polyclonal antibody, rabbit antihuman NF*κ*B polyclonal antibody, and goat antirabbit IgG secondary antibody were obtained from Abcam.

### 2.3. Experimental Method: Immunohistochemistry

The tissues were embedded in paraffin wax and then rinsed with water. The heat antigen repair was done as follows: the specimen was incubated in 3% H_2_O_2_ deionized water for 5-10 min to eliminate endogenous peroxidase activity, rinsed 3 times in PBS for 2 min, then treated with normal goat serum working solution, followed by sealant at room temperature for 10-15 min, prior to an overnight incubation with an appropriate proportion of diluted TNFAIP3 (1 : 200) and NF*κ*B (1 : 400) polyclonal antibody 4°C. Next, the specimen were rinsed thrice with PBS for 2 min each (by dipping in PBS), then treated with biotin-labeled goat antirabbit IgG at 37°C for 10 min, rinsed thrice again in PBS for 2 min each, then treated with HRP-labeled streptomycin working solution (SA/HRP), rinsed again in PBS for 2 min each, then treated with DAB developer, followed by a water rinse, sealing with resin, observation, and filming under light microscope.

### 2.4. Statistical Analysis

Data analysis was conducted using the SPSS 17.0 statistical software. The measurement data are presented as mean standard deviation ($\bar{x}\pm s$). One-way analysis of variance and the LSD-c test were used to conduct pairwise comparisons between the two groups, *n* (%); *P* < 0.05 was considered statistically significant.

### 2.5. Cell Experiment: Data and Methods

#### 2.5.1. Cell of Origin

The CRC cell line, HCT-15, was acquired from the Tianjin Sherwei Biotechnology, Ltd.

#### 2.5.2. Equipment and Reagents

Main equipment: ordinary optical microscope (OLYMPUS Company); fluorescent inverted microscope (OLYMPUS Company); chemiluminescent gel imaging system (Alpha Innotech); transwell chamber (Millipore); RPMI-1640 culture medium (Hyclone); fetal calf serum (Hyclone); protein lysate (Bioworld); protein concentration determination kit (Bioworld); TNFAIP3 polyclonal antibody (Abcam); goat anti-rabbit IgG secondary antibody (Abcam); and TNFAIP3 overexpression lentivirus (Shanghai Genechem Co, LTD).

### 2.6. Experimental Methods

#### 2.6.1. Cell Culture

The lentiviral LV-TNFAIP3 vector, containing the TNFAIP3 gene sequence, was established via reagent to resuscitate CRC cells. The cells were routinely cultured in RPMI-1640 medium containing 10% FBS. While in the log-growth state, viable cells were seeded at 3 × 105 cells/well in 6-well plates and cultured for 24 h until cell confluency reached 70-80%. The cells were then infected with LV-TNFAIP3 (TNFAIP3 group). In the meantime, cells were also infected with the empty vector lentivirus, containing nonspecific sequences, as negative control group (NS group), and additional cells were not treated at all, and served as the blank control group (Con group).

### 2.7. Cell Transfection

The aforementioned 3 groups of cells were maintained inside a 37°C incubator for 12 h, prior to observation under an inverted phase-contrast microscope. In cases where significant cytotoxic effects were evident, the medium was immediately replaced with conventional medium. Otherwise, the transfection was allowed to ensue. After 12-16 h, the transfection medium was replaced with fresh medium, and the cells were incubated at 37°C. Next, the green fluorescent protein (GFP) expression was observed via a fluorescence inverted microscope 72 h posttransfection. Cells exhibiting a transfection rate>80% were used in subsequent experiments. The transfection efficiency (%) was computed as follows: (number of cells expressing GFP/total cells in the same field) × 100%.

### 2.8. CCK8 Detection Method

Following 72 h of transfection, cells in each group were digested with trypsin, and complete medium was used to resuspend the cells, prior to counting. Five 96-well plates were prepared for each group (one plate for each day of proliferation analysis), with 1000 cell/well, and three compound wells with 100 l medium each. Proliferative activity was then detected in each group for 5 consecutive days. Each day beginning on the second day after plate preparation, 10 *μ*l CCK-8 solution was introduced to each well, followed by incubation in an incubator for 2 hours. Absorbance was read at 450 nm using a microplate reader. The data was saved and reevaluated for 5 consecutive days to produce a cell proliferation curve.

### 2.9. Transwell Experiment

The upper chamber surface of the bottom membrane of the transwell chamber was coated with 50 mg/l Matrigel in a 1 : 8 dilution solution, followed by incubation in a 37°C incubator for more than half an hour. The cell suspension was prepared as follows: cells were digested and then resuspended in serum-free medium. Next, 24-well plates were prepared with 3000 cells/per well. 100 l of the prepared cell suspension was introduced to the transwell upper chamber, and 500 l of the FBS medium was introduced to the lower chamber of the 24-well plate. The plate was cultured for 72 h, followed by fixation for 30 min, rinsing with clean PBS, and removal of the upper chamber cells using a cotton swab and subsequent drying. A 20 × Olympus inverted microscope was employed for observation and image capture for analysis.

### 2.10. Western Blotting Experiment

Cell collection was done by adding 200-300 *μ*l RIPA buffer to cells. The protein lysate was placed in an ice bath for 30 min, prior to centrifugation at 12000 g for 15 min. The supernatant was collected and stored at -80°C until protein concentration determination via the BCA kit. Subsequently, the SDS-PAGE glue (10% separation glue and 5% concentrated gel) was used to separate the proteins via electrophoresis. This was followed by film transfer, sealing, primary antibody incubation, wash, secondary antibody incubation, wash, color development, and exposure. Lastly, a chemical exposure machine was employed to capture images for analysis.

### 2.11. Statistical Analysis

All data analyses were done via the SPSS 17.0 statistical software. The measurement data are presented as mean standard deviation ($\bar{x}\pm s$) and were compared using the *T* test. *P* < 0.05 was considered statistically significant.

## 3. Result

### 3.1. The Immunohistochemical Chromogenic Results and Protein Expression Analysis of TNFAIP3 and NF*κ*B Protein Expressions in CRC

**Figure 1:**
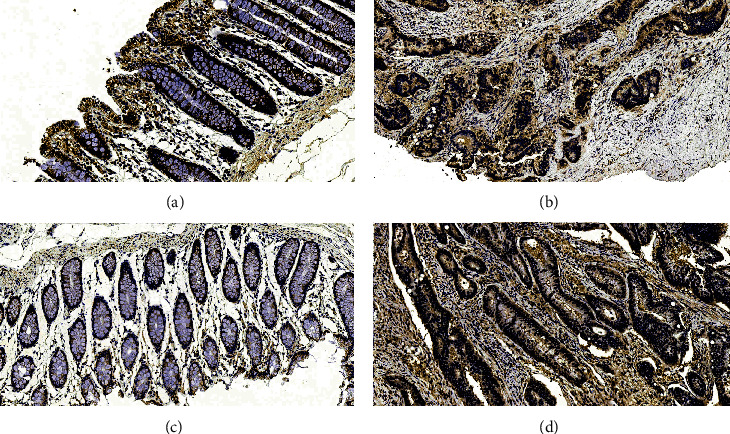
Immunohistochemical color development results.

Figure 1 depicts the immunohistochemical color development results: (a) illustrates the TNFAIP3 protein expression in adjacent tissues, and (b) illustrates the TNFAIP3 protein expression in cancerous tissues, (c) NF*κ*B expression in adjacent tissues, and (d) NF*κ*B expression in cancerous tissues; Table 1 summarizes the expression of TNFAIP3 and NF*κ*B protein expressions in CRC (the positive expression was mainly in cytoplasm).

Based on our results, the NF*κ*B-positive cells were expressed in the cytoplasm of CRC and adjacent tissues. However, but with increasing CRC expression, there was less NF*κ*B expression (*P* < 0.05). There was also reduced TNFAIP3 protein expression in CRC samples, and the difference was significant (*P* < 0.05), as shown in Table 1.

### 3.2. The Relationships between the TNFAIP3 and NF*κ*B Protein Expressions and Clinicopathological Parameters of CRC Patients

The TNFAIP3 and NF*κ*B protein expressions in CRC tissues were not related to sex, age, nerve invasion, and vascular involvement (*P* > 0.05). The TNFAIP3 and NF*κ*B expressions were markedly related to tumor differentiation (*P* < 0.05), lymph node metastasis (*P* < 0.05), and negative correlation (*P* < 0.05) in Table 2.

### 3.3. The Green Fluorescent Protein (Green Fluorescent Protein GFP) Expression Was Observed under an Inverted Microscope

**Figure 2:**
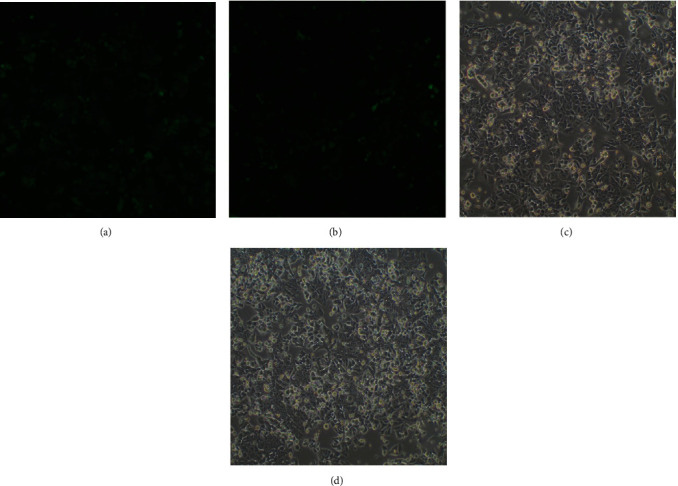
(a) illustrates the NS group; (b) depicts the TNFAIP3 group. In both groups, only cells that demonstrated a transfection rate>80% 72 h after transfection were used to expand the culture for use in subsequent experiments; (c) depicts NS rectal cancer cells, and (d) depicts TNFAIP3 rectal cancer cells.

Live cell morphology and GFP expression were observed under a fluorescence inverted microscope 72 h after transfection. Only cells with a transfection rate>80% were expanded for subsequent experiments. The transfection efficiency (%) was computed as follows: (number of cells expressing GFP/total cells in the same field × 100%), as shown in Figure 2.

### 3.4. CCK8 Test Results

**Figure 3:**
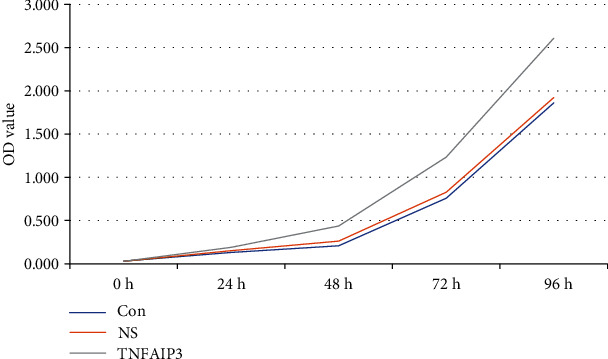
The OD values of the colorectal cancer cells in each group.

The OD values of TNFAIP3 group after 48 h, 72 h, and 96 h were significantly higher than the NS and Con group (*P* < 0.05). In contrast, the OD values of TNFAIP3 group after 0 h and 24 h were not significant than the NS and Con group (*P* > 0.05), as shown in Figure 3.

### 3.5. Transwell Staining Experiment

**Figure 4:**
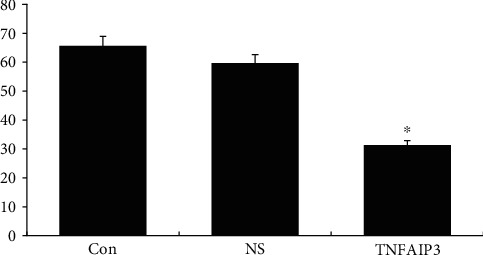
The cell migratory values of the TNFAIP3 cells after 96 h of culture.

**Figure 5:**
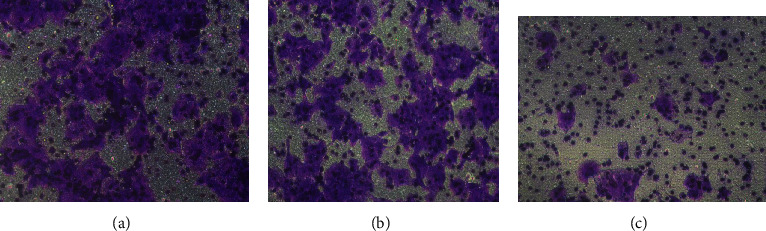
Immunostaining images of TNFAIP3-overexpressing cells after 96 h. (a) depicts Con rectal cancer cells(Con); (b) depicts NS rectal cancer cells(NS), and (c) depicts TNFAIP3 rectal cancer cells(TNFAIP3 group).

The cells, cultured for 96 h, showed significantly less migration and invasion, compared to the NS and Con cells (*P* < 0.05), and there was no significant difference between the NS and Con cells (*P* > 0.05); see Figures 4 and 5.

### 3.6. Western Blotting Experiment Results

**Figure 6:**
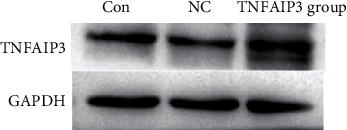
The TNFAIP3 expression in the three groups.

**Figure 7:**
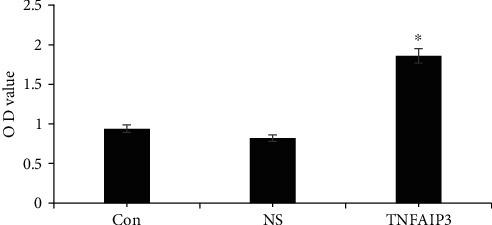
The OD values of the colorectal cancer cells in each group. The TNFAIP3 expression was increased in the rectal cancer cells (TNFAIP3 group), compared to the Con group and NC group.

The TNFAIP3 expression of the three groups were showed in Figures 6. The TNFAIP3 expression was increased in the rectal cancer cells (TNFAIP3 group), compared to the Con group and NC group (*P* < 0.05), as shown in Figures 7.

## 4. Discussion

Chronic inflammation and cancer are closely related in various tissues, and chronic inflammation was reported in multiple tumor samples. CRC risk is highly elevated in patients with ulcerative colitis, and anti-inflammatory drugs are also known to reduce occurrence of intestinal malignancies. Moreover, gastrointestinal tumors are prevalent in patients infected with H. pylori. H. pylori often produces chronic inflammation brought on by the infiltration of various immune cells, including antibody-producing plasma cells, lymphocytes, neutrophils, and macrophages. These cells migrate toward chemokines and inflammatory tissues and act as phagocytes to remove debris and microbes. In response to the inflammatory mediators within tumor tissue, malignant tumor cells also release chemokines that recruit various immune cells to maintain a sustained chronic inflammatory state.

At present, it is speculated that the occurrence and development of CRC is a multifactor, multistage, and multicomplex process. This process activates oncogenes while repressing tumor suppressor genes. TNFAIP3 is a finger domain transcription factor, which is involved in gene expression regulation, cell differentiation, and embryonic development. A prior research demonstrated that the TNFAIP3 expression in a variety of tumors, namely, cervical cancer and liver cancer, is regulated by DNA promoter hypermethylation. Thus far, there are no reported roles of TNFAIP3 in CRC. Therefore, in this study, we explored the expression of TNFAIP3 in CRC tissues and cells. Based on our analysis, the TNFAIP3 protein expression was reduced in CRC tissues, compared to adjacent normal tissues. The NF*κ*B expression in the CRC tissue was not associated with sex, age, nerve invasion, and vascular involvement but was associated with tumor differentiation and lymph node metastasis. The NF*κ*B protein has the most dominant form of the p50/p65 heterodimer, which regulates gene transcription. It was reported that, in the physiological state, NF*κ*B and its repressor protein I*κ*B form an inactive complex. However, under the influence of a variety of inflammatory factors, NF*κ*B protein is activated and transferred to the nucleus. NF*κ*B interacts with the corresponding DNA sites, which can regulate the expression of related target genes (such as TNFAIP3 protein). The TNFAIP3 protein then inhibits the phosphorylation of I*κ*B via multiple different mechanisms, thereby increasing the I*κ*B content, which in turn inhibits NF*κ*B activity [6, 7].

This experimental study revealed that the NF*κ*B protein expression was vastly increased in CRC cells and significantly lower in normal adjacent tissues. This suggested that the NF*κ*B protein is related to CRC. The present experimental data indicated the degree of regulation of the NF*κ*B protein expression within CRC. The lymph node metastases were all significantly associated. The NF*κ*B protein expression in CRC tissues was not associated with patient gender, age, nerve invasion, and vascular involvement. This indicated that the excessive expression of NF*κ*B protein and low expression of TNFAIP3 protein represent a high degree of malignancy. As such, this mechanism may be associated with a tumor-suppressive immune response. It may also be closely related to the occurrence and progression of CRC. Therefore, both these proteins may be used as essential references for determining the prognostic effect of CRC. However, the specific mechanisms underlying the interaction and association of these two molecules on the signaling pathways remain unclear, and further studies are pending. TNFAIP3, otherwise known as A20, is an ubiquitin-editing enzyme that inhibits the NF*κ*B pathway and TNF-induced apoptosis. TNFAIP3 is a key negative regulator of inflammation and immune regulation, and it contains both N (1-385) and C (386-775) terminal regions. The N terminal region has no homology with any known protein structure. Alternately, the C-terminal domain is a zinc-finger region, which gives TNFAIP3 the name zinc-finger protein A20. TNFAIP3 is an important anti-inflammatory molecule that inhibits multiple intracellular signaling pathways.

TNFAIP3 was found to be abnormally expressed in a variety of tumors [8, 9], suggesting its essential role in tumorigenesis and development. The underlying mechanism may be related to its anti- or proapoptotic regulation, which mainly depends on different cell-specific and apoptosis-stimulating factors. In various forms of lymphoma, TNFAIP3 plays a tumor suppressive role, due to inactivated gene mutations, deletions, and promoter methylation [10, 11]. Similarly, in both pancreatic and colon cancers, its expression is reduced [12, 13]. However, its elevated expression is reported in liver cancer, cholangiocarcinoma, and so on.

This study further explored the effect of upregulating TNFAIP3 expression, via Lv-TNFAIP3 lentiviral vectors incorporation, on cell proliferation, invasion, and migration of CRC. The occurrence and development of CRC are regulated by various genes, and it is speculated that TNFAIP3 is an important factor that promotes tumor development. Our results demonstrated that TNFAIP3 upregulation strongly inhibited CRC proliferation, invasion, and migration, therefore signaling that it can be used as a promising therapeutic target for CRC treatment.

## Figures and Tables

**Table 1:** Expression of the TNFAIP3 protein and NF*κ*B protein in CRC tissues.

Metric	TNFAIP3 protein			NF*κ*B protein		
	Positive	Negative	*P*	Positive	Negative	*P*
Experimental group	14 (35.9)	25 (64.1)	0.00	24 (61.5)	15 (38.5)	0.00
Control group	17 (85.0)	3 (15.0)		4 (40.0)	16 (80.0)	

**Table 2:** The relationship between the TNFAIP3 and NF*κ*B protein expressions and the clinicopathological parameters of CRC patients.

Clinicopathological features n		TNFAIP3			NF*κ*B		
		Positive	*X* ^2^	*P*	Positive	*X* ^2^	*P*
*Sex*							
Man	18	6 (33.3)	0.10	0.76	13 (72.2)	0.46	0.50
Woman	21	8 (38.1)			13 (61.9)		
*Age*							
<60 years old	17	8 (47.1)	0.94	0.33	7 (41.2)	1.23	0.27
>60 years old	22	7 (31.8)			13 (59.1)		
*Neural invasion*							
Have	5	1 (20.0)	0.83	0.36	4 (80.0)	0.63	0.43
Not have	34	14 (41.2)			21 (61.8)		
*Pulse duct invasion*							
Have	7	2 (28.6)	0.20	0.66	6 (85.7)	1.73	0.19
Not have	32	12 (37.5)			19 (59.4)		
*Degree of differentiation*							
Middle and low	24	12 (50.0)	5.39	0.02	16 (66.7)	10.57	0.00
Tall	15	13 (86.7)			2 (13.3)		
*Type*							
Ulceration type	32	12 (37.5)	0.07	0.79	22 (68.8)	3.92	0.05
Protrude type	7	3 (42.9)			2 (28.6)		
*Transition*							
Have	28	7 (25.0)	7.12	0.02	23 (82.1)	5.25	0.02
Not have	11	7 (63.6)			5 (45.5)		

## Data Availability

The data used to support the findings of this study are available from the corresponding author upon reasonable request.
